# Rapid tissue regeneration induced by intracellular ATP delivery—A preliminary mechanistic study

**DOI:** 10.1371/journal.pone.0174899

**Published:** 2017-04-05

**Authors:** Harshini Sarojini, Adrian T. Billeter, Sarah Eichenberger, Devin Druen, Rebecca Barnett, Sarah A. Gardner, Norman J. Galbraith, Hiram C. Polk, Sufan Chien

**Affiliations:** Department of Surgery, University of Louisville, Louisville, Kentucky, United States of America; Kyoto Daigaku, JAPAN

## Abstract

We have reported a new phenomenon in acute wound healing following the use of intracellular ATP delivery—extremely rapid tissue regeneration, which starts less than 24 h after surgery, and is accompanied by massive macrophage trafficking, in situ proliferation, and direct collagen production. This unusual process bypasses the formation of the traditional provisional extracellular matrix and significantly shortens the wound healing process. Although macrophages/monocytes are known to play a critical role in the initiation and progression of wound healing, their in situ proliferation and direct collagen production in wound healing have never been reported previously. We have explored these two very specific pathways during wound healing, while excluding confounding factors in the in vivo environment by analyzing wound samples and performing in vitro studies. The use of immunohistochemical studies enabled the detection of in situ macrophage proliferation in ATP-vesicle treated wounds. Primary human macrophages and Raw 264.7 cells were used for an in vitro study involving treatment with ATP vesicles, free Mg-ATP alone, lipid vesicles alone, Regranex, or culture medium. Collagen type 1α 1, MCP-1, IL-6, and IL-10 levels were determined by ELISA of the culture supernatant. The intracellular collagen type 1α1 localization was determined with immunocytochemistry. ATP-vesicle treated wounds showed high immunoreactivity towards BrdU and PCNA antigens, indicating in situ proliferation. Most of the cultured macrophages treated with ATP-vesicles maintained their classic phenotype and expressed high levels of collagen type 1α1 for a longer duration than was observed with cells treated with Regranex. These studies provide the first clear evidence of in situ macrophage proliferation and direct collagen production during wound healing. These findings provide part of the explanation for the extremely rapid tissue regeneration, and this treatment may hold promise for acute and chronic wound care.

## Introduction

Wound healing is a complex and dynamic process involving the replacement of devitalized and missing structures. The traditional view of wound healing is that it involves hemostasis, inflammation, proliferation, and remodeling, and these steps result in a lag of 3–6 d before reepithelialization starts [1,2]. We have discovered that the intracellular delivery of adenosine triphosphate using ATP-vesicles as an acute wound treatment enhances wound healing [3,4]. The most unprecedented finding was that new tissue started to generate within 24 h, and it continued to grow to eliminate the wound cavity quickly [4–6]. This growth was attained by early and massive monocyte/macrophage trafficking, proliferation, and fast collagen production for direct formation of extracellular matrix (ECM). Reepithelialization tunneled through the granulation tissue [4] and the upper surface of the granulation tissue eventually fell off, revealing a perfectly healed wound. More importantly, the granulation tissue growth had a self-limiting feature, so that no hypertrophic scar formation or any other unusual growth was evident, even after two years [4,6]. This healing process is totally different from the conventional wound healing process, where fibrin, platelets, and red blood cells serve as the main components of the early provisional matrix, which is gradually replaced by granulation tissue during the proliferation phase after a lag of 3–6 d [1,7]. The ATP-vesicle triggered healing process therefore essentially eliminates the traditional lag time and significantly shortens acute wound healing times.

The mechanisms for the extremely rapid ECM formation following intracellular ATP delivery are entirely unclear because no one has ever reported any similar process in the past. Traditionally, the provisional ECM differs in composition and function from the ECM found in normal tissue, in which platelet aggregation forms a hemostatic plug and blood coagulation forms the provisional matrix. The resulting densely cross-linked network then prevents excessive blood loss [8]. The components also include fibronectin, collagen, glycosaminoglycans, elastin, glycoproteins, and proteoglycans [9]. The platelets in the matrix release growth factors and adhesive proteins that stimulate the inflammatory response. This induces cell migration into the wound and the cells use the provisional matrix as a substrate [8,10].

The provisional matrix is gradually replaced by granulation tissue, which begins to appear shortly after wounding and functions as rudimentary tissue that continues to grow until the wound bed is covered. The newly formed granulation tissue consists of new blood vessels, fibroblasts, inflammatory cells, endothelial cells, myofibroblasts, and collagen; these form the basis for reepithelialization. The normal ECM is a dynamic macromolecular complex synthesized primarily by fibroblasts, which assemble into a network that surrounds cells. Fibroblasts normally migrate to the site of damage following injury and produce collagen to facilitate healing.

The whole wound recovery process seems totally different in our ATP-vesicle treated wounds [4–6] The key features are as follows: 1) the regeneration of tissue begins at an extremely early time point—something never seen or reported before; 2) this early regenerated tissue does not resemble the traditional provisional matrix that has to be replaced gradually by granulation tissue; 3) the main component of the very early regenerated tissue is monocytes/macrophages, and these cells show highly proliferative features; 4) these macrophages seem to produce collagen directly because histological staining of these wound tissues show no or very few fibroblasts in the granulation tissues; and 5) reepithelialization, which does not occur in the provisional matrix, occurs as early as day 3 in the ATP-vesicle treated wounds, as new epithelial tissues tunnel through the solid granulation tissue and heal the wound without scar formation. These features are very unconventional and perhaps difficult to believe. However, they have been seen in ear wounds from more than two hundred adult rabbits (over 1600 wounds) following treatment with ATP-vesicles. Although rabbits have the ability to regenerate ear cartilage when the perichondrium is preserved, their skin regeneration capacity is similar to that seen in other mammals[11,12]. Numerous dressings have been tested thousands of times on wounds in rabbits, but none has produced the rapid tissue regeneration seen in our studies.

The mechanisms behind this unprecedented phenomenon are complex and we have provided some preliminary explanations in our previous publications [4–6]. However, delineating individual factors in the in vivo environment is challenging because multiple interacting factors come into play. The current study has two purposes: 1) to provide additional in vivo findings related to the unprecedented ATP-vesicle driven wound healing phenomenon to support our hypothesis; and 2) to use in vitro cell culture to confirm some of the in vivo findings in the absence of other confounding factors. Through continuous explorations, we hope to ultimately discover some of the critical mechanisms that are involved in the extremely rapid tissue regeneration observed in these treated wounds and to incorporate this new knowledge to improve wound management strategies.

## Materials and methods

### Materials

The ATP-vesicles were provided by Avanti Polar Lipids Inc. (Alabaster, AL) and provided to us in a freeze-dried form [5]. The ATP-vesicles were reconstituted with normal saline or 1640 RPMI medium (MP Biomedicals, Solon, OH) before use. After reconstitution, the composition was: 100 mg/ml of soy phosphatidylcholine (Soy PC)/1, 2-dioleyl-3-trimethylammonium-propane (DOTAP) (50:1), trehalose/Soy PC (2:1), 10 mM KH_2_PO_4_, and 10 mM Mg-ATP. The diameters of the lipid vesicles were evaluated with a Shimadzu SALD-7500 Nano Particle Size Analyzer (Shimadzu Scientific Instruments, Columbia, MD) and ranged from 85 to 150 nm.

Recombinant human platelet-derived growth factor-BB (rhPDGF-BB, becaplermin topical gel, Regranex) was made by Healthpoint Biotherapeutics (Fort Worth, TX). Mg-ATP was purchased from Sigma-Aldrich (St. Louis, MO), human CD14-FITC and human HLA-DR-PE antibodies were purchased from BD Bioscience (San Jose, CA), Anti-MAC387 was from AbD Serotec (Raleigh, NC), Anti-Collagen 1 antibodies were from Abcam (Cambridge, MA).

### Animals and wound creation

The in vivo animal study was conducted in accordance with the National Institutes of Health guidelines for the care and use of animals in research and the protocol was approved by the Institutional Animal Care and Use Committee of the University of Louisville, an AAALAC accredited program. A total of 27 adult New Zealand white rabbits (2.0–3.0 kg, Myrtle’s Rabbitry, Thompson Station, TN; and Harlan Laboratories, Indianapolis, IN) were used. Among them, 9 rabbits (72 wounds) were used for reepithelialization and granulation tissue growth comparison. The remaining 18 rabbits were sacrificed for histologic and immunohistochemistry studies. Three rabbits each were used for Regranex treatment comparison for 1, 3, 4, and 15 days. Three rabbits each were used for treatment with normal saline comparison for 4, and 15 days. In each rabbit, 8 wound samples were taken (4 for ATP-vesicles and 4 for Regranex/saline) for the analyses after sacrificing.

Wounds were created using a minimally invasive technique developed in our laboratory, as reported before [13,14]. Briefly, under general anesthesia (ketamine 50 mg/kg and xylazine 5 mg/kg, IM), one ear was rendered ischemic by a minimally invasive technique. Four full-thickness skin wounds (6 mm in diameter) were made to the depth of the cartilage on the ventral side of each ear (8 wounds for both ears) with a stainless steel punch. The perichondrium was removed with the skin or separately. The base of the wound consisted of cartilage but the cartilage itself was not perforated. Postoperatively, all animals received analgesics in the form of a fentanyl patch (25 μg/h) and buprenorphine (0.01 mg/kg, IM) for 2–3 d to reduce possible pain. The animals were allowed free access to food and drink.

The wounds were treated with two types of dressings: The two wounds on one side of the ear were treated with ATP-vesicles (10 mM Mg-ATP), while the control wounds on the other side of the same ear received Regranex/Saline. We did dose-response studies previously and found Mg-ATP concentrations in the ATP-vesicles at the range of 5–20 mM to be the best in wound care. Regranex is a ready-made gel with the concentration of 0.01% which has been tested in numerous preclinical and clinical studies and there was no clinical benefit when increasing the concentration above 0.01% [15]. The dressing volume was dynamic according to wound size: Immediately after surgery, about 0.5 ml was needed for each wound. The volume decreased gradually when the wound size is reduced over time. The wound was covered with TegaDerm^TM^ (3M, Minneapolis, MN) to prevent desiccation. Dressings were applied once a day and changed every day. The old dressings were removed and the wounds were cleaned with cotton swabs to remove any fluid, clots, fibrin, residual drugs, and any tissue debris. Digital photos were taken, new dressings were applied, and the wounds were covered again with TegaDerm^TM^. This procedure was performed until all wounds were healed or rabbits were sacrificed. For the BrdU Labelling studies, the BrdU reagent was injected intraperitoneally (10 ml concentrated reagent/ kg body weight) 24 h prior to sacrifice.

### Wound healing measurements

Digital images were taken daily with scales until the wounds were totally healed. Wound reepithelialization was measured by tracing the epithelialized areas from the wound edge towards center. The remaining non-epithelized area was compared with the original wound area to obtain the healing rate by morphometric analysis using the NIH image software ImageJ [16,17].

### Histology and immunohistochemical analyses

For histology studies, rabbits were sacrificed at different days post-surgery with an overdose of KCl (1–2 mmol/kg, IV) when they were under deep anesthesia (ketamine 100 mg/kg+xylazine 10 mg/kg, IV). Wound samples were taken with a circular punch to include both the wound and surrounding tissue (a 2–3 mm ring around the wound), and were fixed in 4% buffered formaldehyde and embedded in paraffin for simple histologic study. For immunohistochemical studies, fresh samples were prepared or with antibodies injected before sacrifice (such as BrdU). The paraffin blocks were cut in 5 μm slices and mounted on Superfrost Plus microscopic slides (Fisher Scientific, Pittsburgh, PA). The serial sections were further analyzed by immunostaining (5 μm thickness) on the slide. Sections were deparaffinized with xylene and rehydrated through a graded alcohol series, ending with Tris buffered saline containing Tween 20 (Fisher Scientific, Pittsburgh, PA). Antigen retrieval was done at 100°C in 10 mM Tris-HCl buffer (Fisher Scientific, Pittsburgh, PA) for 1 min. The endogenous peroxidase activity was quenched by incubation with 0.3% H_2_O_2_ (Fisher Scientific, Pittsburgh, PA) and non-specific antibody binding was blocked with RTU Serum-free Protein Block (Dako, Carpinteria, CA) for 20 min. The slides were then incubated with primary antibody at 37°C for 1 h. Anti-proliferating cell nuclear antigen (PCNA) was purchased from Santa Cruz Biotech (Dallas, TX), and anti-BrdU antibody was purchased from Millipore (Billerica, MA). These were subsequently incubated with RTU EnVision+ HRP Labelled Polymer anti-mouse second antibody (Dako, Carpinteria, CA) for 30 min at 37°C. The sections were rinsed after each reaction. Finally, the immunoreaction products were visualized with a solution of DAB+ (Dako, Carpinteria, CA). Imaging was done using a Nikon Eclipse Ti fluorescence microscope (Nikon Instruments Inc., Melville, NY). Collagen staining was accomplished with Picrosirius red (Polysciences, Inc., Warrington, PA) staining followed by circular polarized microscopy (Carl Zeiss, Thornwood, NY) to evaluate the different forms of collagens in the wound site. The yellow to orange color was quantified using NIS Elements software (Nikon Instruments Inc., Melville, NY).

### Cell preparation and culture

Human primary monocyte/macrophages and RAW 264.7 (sigma) cells were used for this study. The protocol for primary macrophage cell culture studies was approved by the University of Louisville Institutional Review Board prior to enlisting any study subjects (HSPPO 08.0018). Written informed consent was obtained from all participants. A total of 16 healthy donors were enrolled; the majority of the experiments were conducted with seven donors per experiments. The age of the participants ranged from 19–49 years. Venous blood was collected in EDTA Vacutainers (Becton Dickinson, Franklin Lakes, NJ). Primary human monocytes were isolated using the magnetic positive selection technique according to manufacturer’s instruction. Briefly, the whole blood was incubated with Human CD14 MicroBeads (Miltenyi Biotec, Auburn, CA) at 37°C in a CO_2_ incubator for 15 min. After washing, the blood was resuspended to the original volume using MACS Separation Buffer (Miltenyi Biotec, Auburn, CA) and then run through Whole Blood Magnetic Columns (Miltenyi Biotec, Auburn, CA). After isolation, the columns were washed three times and the monocytes were eluted from the columns with MACS Elution Buffer (Miltenyi Biotec, Auburn, CA). The cells were washed twice with phosphate buffered saline (PBS) and counted. The purity of the isolated monocytes was >95% as determined by flow cytometry.

We tested the concentration of ATP-vesicles for cell culture in the past and found a concentration of 0.5 mM to 1 mM to be the best for cell culture.[18,19]. Regranex gel dilutions in media has been tested in cell culture and the concentration below 10 μg (0.001%) did not show any significant response in our experiments [20].

The primary monocytes were collected and cultured in 1640 RPMI medium (MP Biomedicals, Solon, OH) supplemented with 10% heat-inactivated fetal bovine serum, 2 nM L-glutamine, 100 IU/ml penicillin, 100 μg/ml streptomycin and 250 ng/ml amphotericin B (Thermo Scientific, Waltham, MA). The monocyte cells were plated in 24-well culture plates at a concentration of 0.5 x 10^6^ cells/ml/well in a humidified incubator with 5% CO_2_ at 37°C. The human monocytes/macrophages and the RAW 264.7 cells were treated 24 h later with 1 mM ATP-vesicles, 1 mM free Mg-ATP, 1 mM empty lipid vesicles, or 0.001% Regranex as controls for another 24 to 48 h. After treatment for the indicated times, the cell culture supernatant was collected and stored at -80°C until measurement.

### Flow cytometry analysis

After isolation of the human monocytes as described above, 1 x 10^5^ monocytes were prepared for subsequent analysis by flow cytometry. After undergoing a washing step using phosphate buffer saline (PBS, Sigma-Aldrich, St. Louis, MO), the cells were stained with human CD14-FITC and human HLA-DR-PE antibodies for 25 min at 4°C according to manufacturer’s instruction. After staining, remaining red blood cells were lysed with hypotonic lysis buffer for 6 min at 4°C. Following lysis, the monocytes were washed with PBS and then fixed in 1% paraformaldehyde (Polyscience Inc., Warrington, PA). Cells were immediately acquired using a FACSCalibur (BD Bioscience, San Jose, CA). Data were analyzed using Cell Quest Software (BD Bioscience, San Jose, CA).

### Hematoxylin eosin staining and immunocytochemical analysis

Cells were fixed with 10% formalin and stained with Hematoxylin and eosin to examine the morphological changes in the cells in response to different treatments. For immunocytochemistry, the cells were fixed with 10% formalin and permeabilized with 0.1% Triton X-100 (Fisher Scientific, Pittsburgh, PA). The cells were incubated with primary antibody for 1 h at 37°C and then incubated with FITC-conjugated Goat anti mouse secondary antibody (Invitrogen, Carlsbad, CA) for 1 h at 37°C. The cells were mounted using Permount mounting solution (Fisher Scientific, Pittsburgh, PA). Fluorescence was detected using a fluorescence microscope Nikon Eclipse Ti microscope (Nikon Instruments Inc. Melville, NY).

### Enzyme-linked immunosorbent assays

Collagen type 1 alpha 1 levels were determined in the culture supernatant by enzyme-linked immunosorbent assays (ELISA) (My Biosource LLC, San Diego, CA) in 96-well plates according to manufacturer’s protocol. All samples were analyzed in duplicate; collagen levels of samples were determined from a standard curve prepared using the collagen standard provided in the kit. Levels of IL-10, TNF alpha, IL-6, and MCP-1 in the culture supernatants were measured using ELISA kits (eBioscience, Inc. San Diego, CA) according to the manufacturer’s protocols. Cytokine and chemokine levels of samples were determined from a standard curve prepared using recombinant human IL-10 TNF alpha, IL-6, and MCP-1. Absorbance was determined at 450 nm on a micro plate reader (Multiskan MCC/340, Fisher Scientific, and Pittsburgh, PA). The results were compared with a standard curve prepared from titrated standards.

### Sircol collagen assay

The manufacturer’s protocol was followed for Sircol collagen assays. Briefly, 100 μl of cell culture supernatant was added to 1 ml of the colorimetric reagent (the dye SR in picric acid) and vortexed for 45 min, followed by centrifugation at 10,000g for 10 min. The SR dye was released from the pellet with alkali reagent (1 N NaOH) and absorbance was determined at 540 nm on a micro plate reader (Multiskan MCC/340, Fisher Scientific, Pittsburgh, PA). Absolute values were attained with a standard graph composed of the collagen type 1 standard supplied with the kit, in the range of 5–100 μg per 0.1 ml

### Statistical analysis

Results are expressed as the mean ± standard deviation (SD). All tests were performed using SPSS Professional Edition software (IBM). Descriptive statistics, including mean and SD, along with two tailed T tests, were used to determine significant differences. *P* values less than 0.05 were considered significant.

## Results

### Wound reepithelialization comparisons

**Fig 1:**
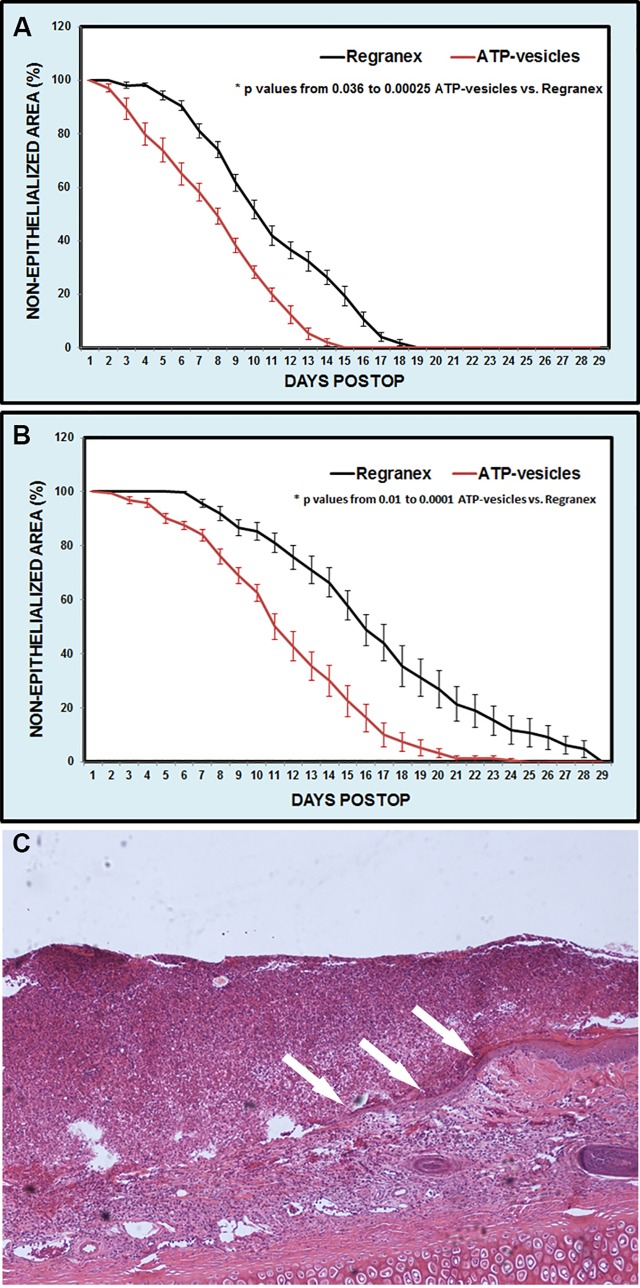
Comparison of wound reepithelialization between ATP-vesicles and Regranex. When 50% of wound was reepithelialized in the non-ischemic wounds, it took an average of 8 days for ATP-vesicles while 10.5 days for Regranex (p = 0.00026, **1A**). In the ischemic wounds, the average 50% reepithelialization time for ATP-vesicles was 11.2 days vs. 16.1 days for Regranex (p = 0.0001, **1B**). Because reepithelialization often tunnels through the granulation tissue in the wounds treated by ATP-vesicles (**1C**), the actual reepithelialization in these wounds might be faster.

**Fig 2:**
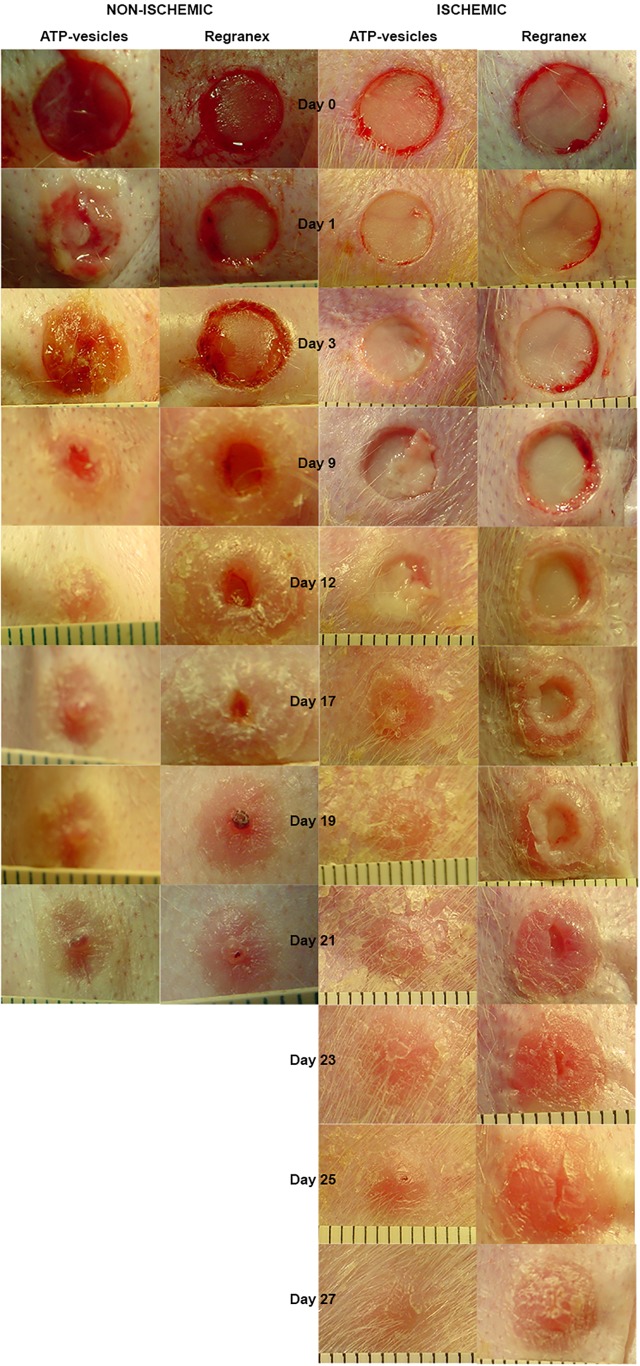
A representative comparison of wound granulation and healing between the two groups. In the wounds treated by ATP-vesicles, new tissue starts to appear only 24 hours after surgery in the non-ischemic wounds. In the ischemic wounds, generation of granulation tissue is slower. However, the wound still heals faster than that treated by ATP-vesicles.

Wound reepithelialization was enhanced by the ATP-vesicle treatment. On the non-ischemic ears, the average time for complete reepithelialization was 12.06 ± 1.45 days (mean±SD) for ATP-vesicle-treated wounds versus 15.72 ± 1.45 days for Regranex-treated wounds (n = 18, p = 0.00043, Fig 1A). On the ischemic ears, the average complete reepithelialization time for ATP-vesicles was 16.00±3.39 days vs. 21.61±4.30 days for Regranex-treated wounds (p = 0.0001, Fig 1B). Due to the rapid granulation tissue regeneration in the wounds treated by ATP-vesicles, reepithelialization often tunneled through the granulation tissue while the top was still covered by the granulation tissues (Fig 1C). As such, the actual reepithelialization rate in the wounds treated with ATP-vesicles might be faster. Fig 2 is a representative healing comparison between the two groups in both ischemic and non-ischemic wounds. The early granulation tissue appears pale, edematous, and fragile due to a lack of early neovascularization. However, it gradually solidifies and becomes pink looking after 2–3 days when newly formed vascular supply is established [4–6].

### Macrophage in situ proliferation

**Fig 3:**
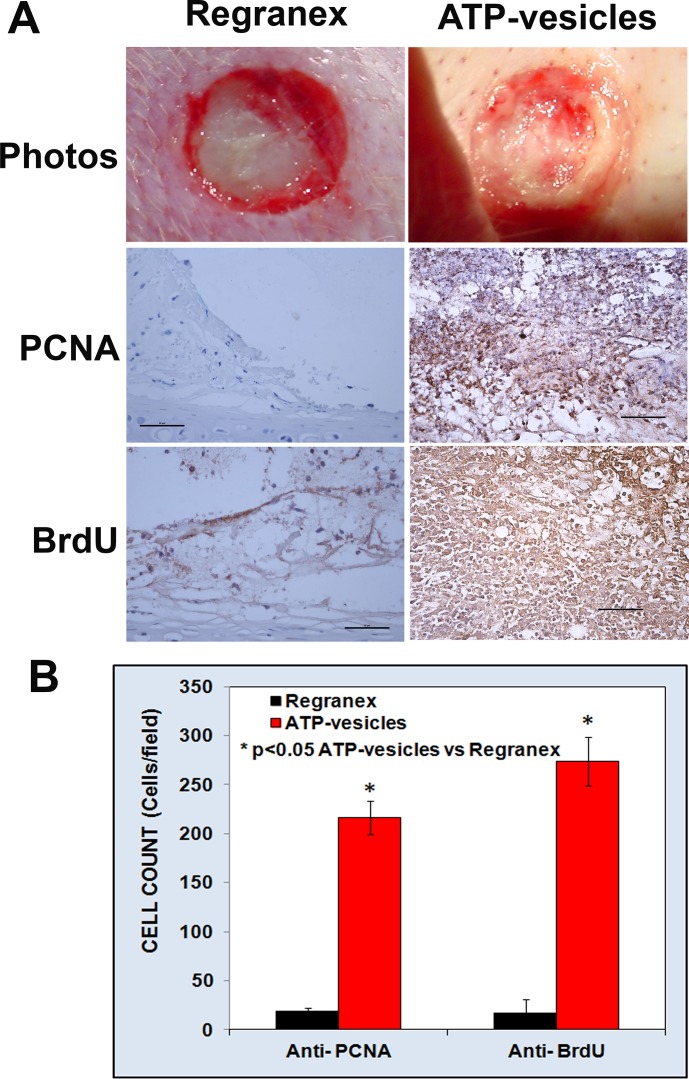
Wound cell comparison at day 1 after treatment. **(A)**: The wound treated with ATP-vesicles has already been covered by newly generated tissues. Anti-PCNA and BrdU stains show very actively proliferating cells in ATP-vesicle treated wounds when compared to Regranex treated wounds. The brown color indicates DAB-positive immunostaining (scale bar = 50 μm). **(B)**: Bar graph showing the differences between the two groups.

Proliferating Cell Nuclear Antigen (PCNA) and BrdU immunostaining showed significantly greater numbers of PCNA- and BrdU-positive cells early in the wounds treated with ATP-vesicles when compared to the controls (Fig 3, scale bar = 50 μm). The highest macrophage accumulation occurred at 24 h, in which nearly tenfold more macrophages were present in the wounds treated with ATP-vesicles than in wounds treated with Regranex [6]. The control wounds treated with Regranex showed no equivalent early tissue growth or spike in macrophage numbers, but the numbers of macrophages increased somewhat after day 3, as is typically reported in the literature for the normal wound healing reactions [1].

### Enhanced collagen production

**Fig 4:**
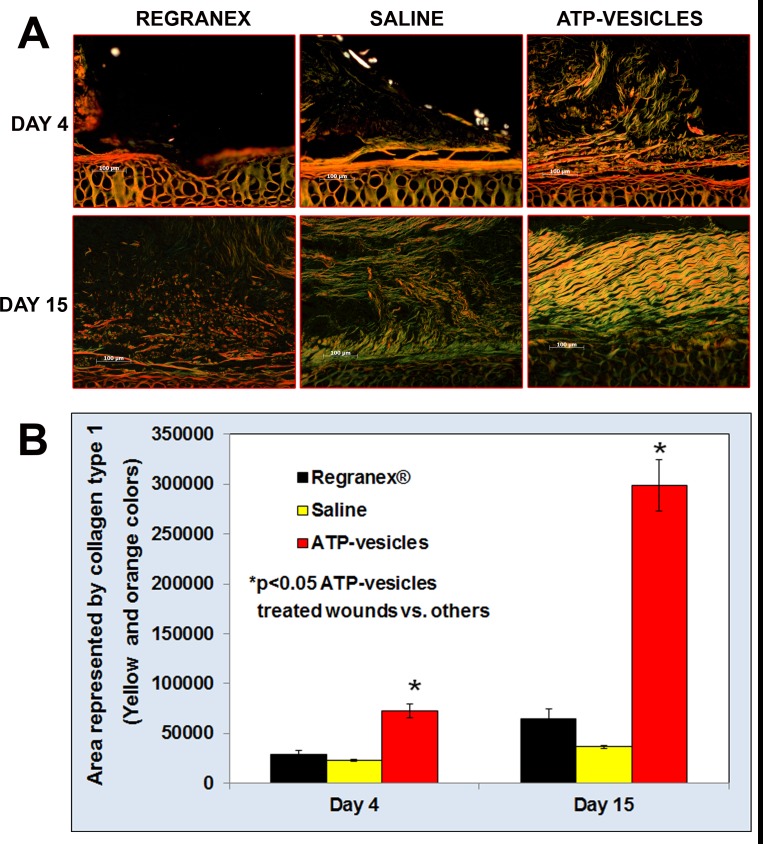
Comparison of the picrosirius red staining at days 4 and 15. **(A):** The wound treated with ATP-vesicles shows a higher volume of collagen (bright red), while the control wounds have little collagen. The picrosirius red staining is viewed under circular polarized light. Increased type I (orange) and type III collagen (green) content is seen in ATP-vesicle-treated wounds as healing progresses. The control wounds have much less collagen expression. The collagen fibers also increase in size and mature at a faster rate in ATP-vesicle-treated wounds when compared with the controls (Scale bar is 100 μm). **(B)**. Graphical representation of morphometrically measured area represented by collagen type 1 among the three groups.

Massive cell accumulation alone in a wound cavity does not constitute real healing since a loose cell mass could die or dislodge easily. However, the cell mass in the wound cavity in our study was firmly connected to the wound edge or base, suggesting the enhanced production of connective tissues. Van-Gieson staining revealed thicker fibrillar collagen assembly near the wound edge in ATP-vesicle-treated wounds [6]. Further analysis by circular polarizing microscope of the wound collagen stained by picrosirius red indicated thicker type I (orange to red color, more mature), type II (yellow), and some type III (green, newly formed) collagen fibrils in the wounds treated with ATP-vesicles, while the wound treated with saline and Regranex^®^ had less and weaker collagen organization (Fig 4A). Starting from days four to fifteen, a gradual accumulation and thickening of fibrillar collagen was evident in the ATP-vesicle-treated wounds while the wounds treated with saline and Regranex had less accumulation of type 1 collagen (Fig 4B).

### Isolation and characterization of human monocytes

**Fig 5:**
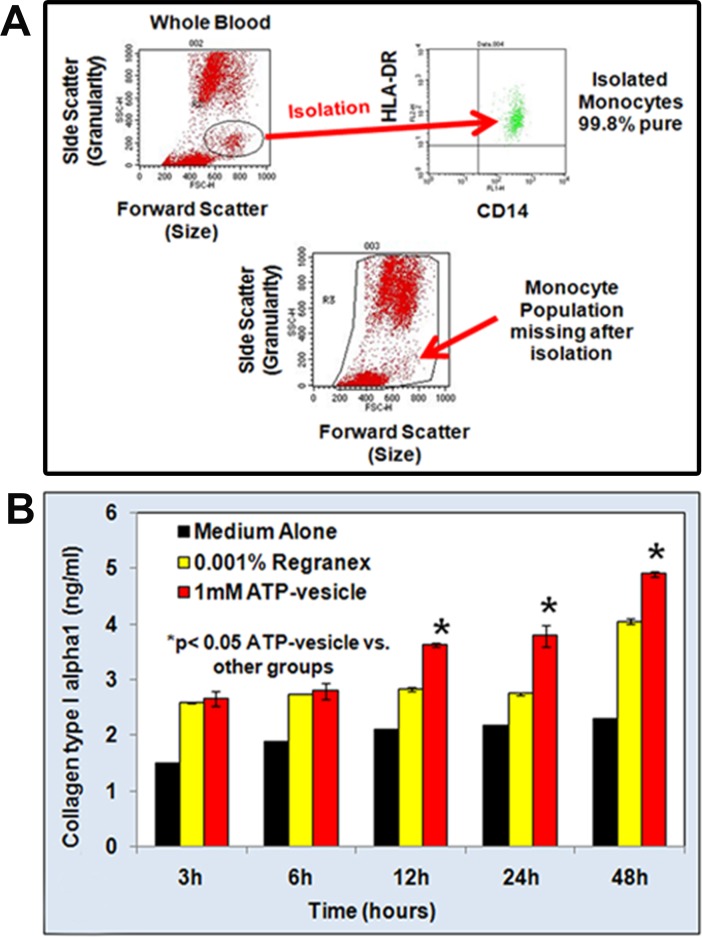
**(A) FACS analysis to determine the purity of monocytes isolated from human blood.** The isolated cells were CD-14 and HLA-DR positive. The purity of the isolated cells was 98%. **(B): Time response effect of various treatments on collagen production in human macrophages**. *p<0.05 ATP-vesicles vs. Regranex and medium. The monocyte/macrophage (500,000 cells/ml) cells were treated with ATP-vesicles (1 mM Mg-ATP) and controls for 3, 6, 12, 24, and 48 h. The collagen type 1α1 levels increased with time when the macrophages were treated with 1mM ATP-vesicles when compared with macrophages treated with Regranex or culture medium alone.

Primary monocytes were isolated from human blood. Initial isolation experiments were carried out by positive selection using CD14 antibodies to monocytes. The purity of these isolated monocytes was then determined by FACS using CD14 and HLA-DR antibodies. A total of 100,000 cells were used for each experiment. FACS analysis of these cells showed 99.8% CD14 and HLA-DR positivity. This was further confirmed by cell sorting of whole blood before and after the isolation of CD14 monocyte cells (Fig 5A). The population of the CD14 positive cells was absent from the three-part differential of leukocytes after isolation.

### Direct collagen synthesis by macrophages when treated with ATP- vesicles

Collagen type 1 ELISA studies confirmed the increased release of collagen into the culture supernatant with increasing doses of ATP-vesicles [6] and culture time. The monocytes were allowed to attach to the plates and were then treated with ATP-vesicles containing different concentrations of Mg-ATP. The resulting collagen release was both dose- and time-dependent: The monocytes/macrophages released increasingly higher levels of collagen as the Mg-ATP concentration increased from 1 mM to 10 mM [6], and as the culture period increased in duration. However, treatment with free Mg-ATP or Regranex did not increase collagen type 1α1 production. The cells treated with all the components of ATP-vesicles survived less than 3 d, a much shorter duration than observed with cells treated with ATP-vesicles (data not shown). Based on these results, we selected ATP-vesicles containing 1 mM Mg-ATP for all subsequent in vitro human macrophage experiments.

Human macrophages were then treated with ATP-vesicles (containing 1 mM Mg-ATP) for 48 h, and compared with medium alone or Regranex as control treatments. Collagen levels in all the three treatments were measured at 3, 6, 12, 24, and 48 h. Collagen release was increased significantly in ATP-vesicle treatments with increasing time, starting from 12 h. However, Regranex treatment also caused an increase in collagen, but only at 48 h (Fig 5B), in agreement with our previous in vivo findings [6].

### Immunocytochemical studies

**Fig 6:**
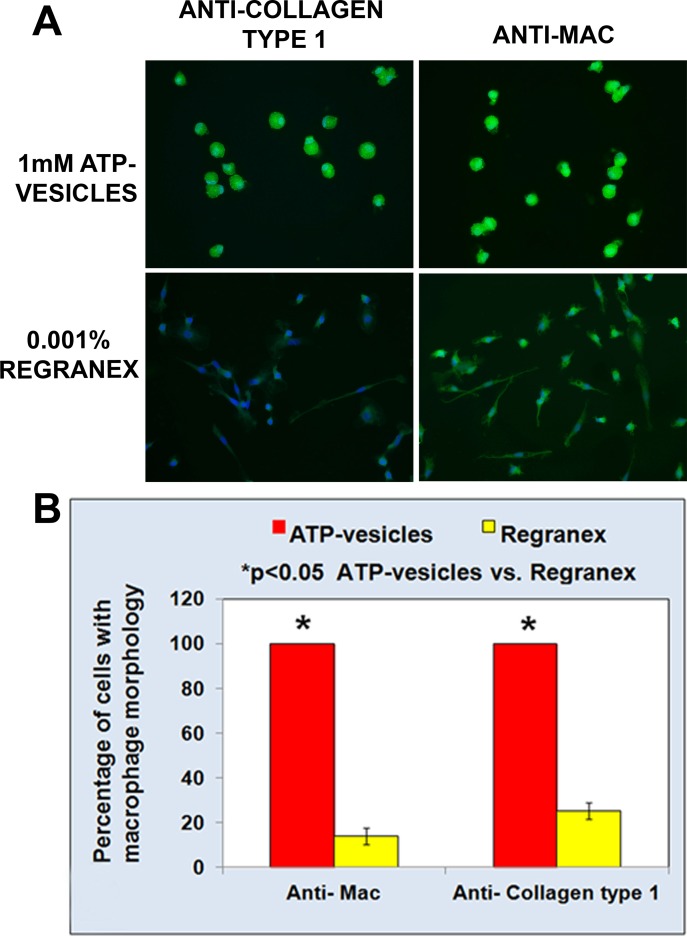
**(A): Anti-mac387 and collagen type 1α1 immunostaining on monocyte/macrophage cells treated with 1 mM ATP-vesicle or Regranex for 5 d**. Cells treated with medium alone didn’t survive for 5 d. Green color represents anti-mac or type 1α1 collagen staining and blue is the DAPI staining for the nucleus. Collagen type 1α1 immunostaining is higher in ATP-vesicle treated cells when compared with the controls (scale bar = 50 μm). **(B)**. Graphical representation of the cells having macrophage phenotype following treatments.

Macrophages are known to undergo rapid transformation into fibroblasts and the traditional view is still that fibroblasts are the major source of collagen. Thus, confirmation of macrophage phenotype during collagen production becomes critical in our hypothesis. For this reason, we used double-immunostaining to obtain further confirmation of the cell phenotypes and their relationship with collagen production. The cultured cells were first immunostained with anti-mac 387 antibodies to prove that they were indeed macrophages, and later stained with an anti-collagen type 1 antibody to show they can release collagen. On day five, the ATP-vesicle-treated cells still maintained their macrophage phenotype and antigenic activity and showed high collagen expression; while the cells treated with Regranex already showed a fibroblast-like morphology with much less anti collagen type 1 antigenic activity (Fig 6). These results clearly confirm that: 1) intracellular ATP delivery extends in vitro macrophage survival time, and 2) even in the in vitro environment, macrophages can produce collagen directly without first transforming into fibroblasts.

### Cytokine production

**Fig 7:**
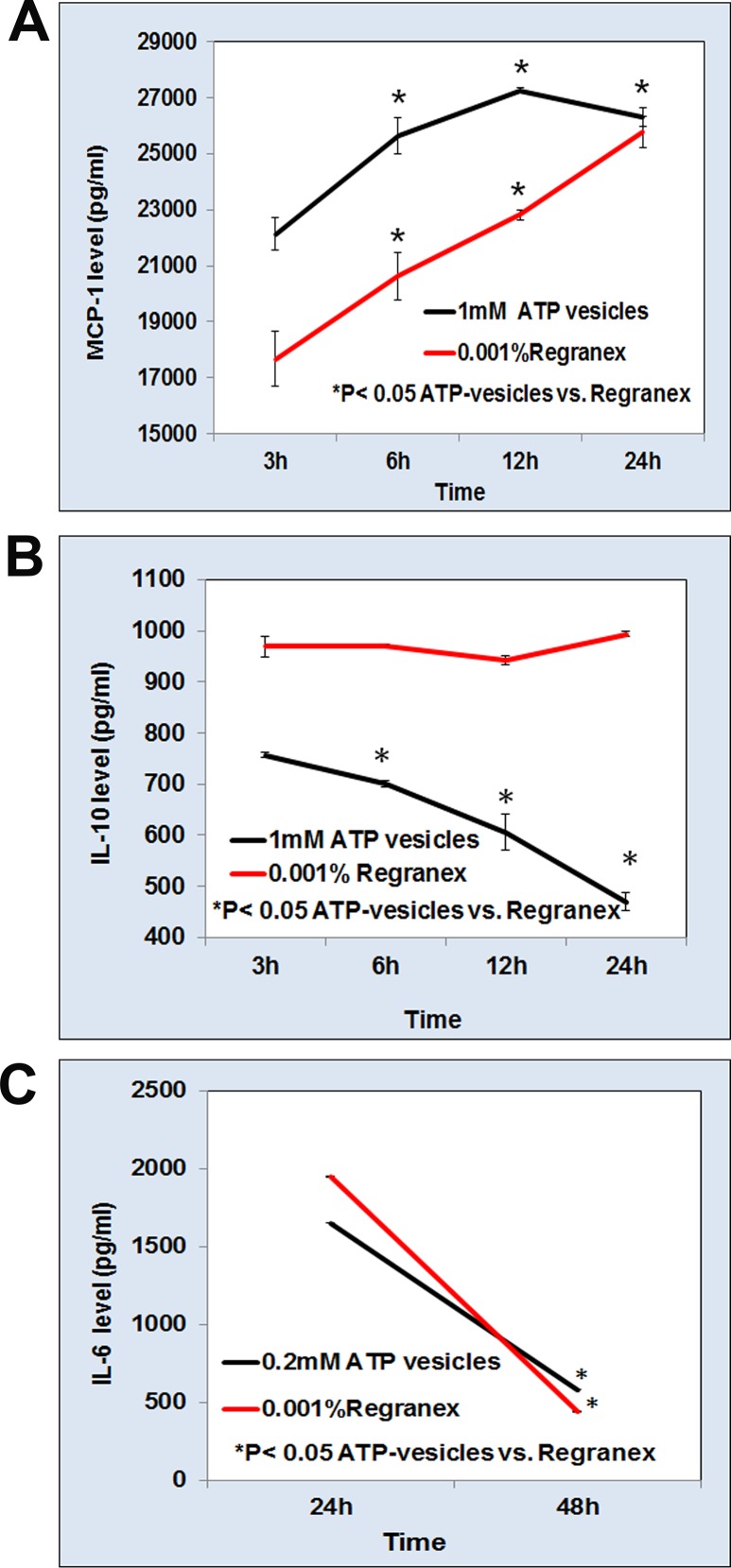
Effect of ATP-vesicles and Regranex on MCP-1, IL-10 and IL-6 production in cultured human monocyte/macrophages. **(A-C)**: The release of MCP-1, IL-10 and IL-6 in culture supernatant was measured by ELISA method. Data are the mean ± SD of six independent experiments (*p<0.05).

We explored the roles of cytokines and chemokines in the very rapid healing process by also testing several cytokines/chemokines that are known to play major roles in wound healing. The monocyte/macrophage cells (500,000 cells/ml) were cultured under standard conditions and then treated 24 h later with ATP-vesicles (1 mM Mg-ATP) or 0.001% Regranex for different times. The release of monocyte chemotactic protein I (MCP-1), interleukin-10 (IL-10), and IL-6 into the culture medium was measured by ELISA, which confirmed an enhanced MCP-1 production with increasing culture time. However, treatment with ATP-vesicles resulted much higher MCP-1 expression than with Regranex (Fig 7A). Regranex treatment enhanced IL-10 production while ATP-vesicle treatment reduced IL-10 production (Fig 7B). IL-10 is an immunomodulatory cytokine known to regulate the inflammatory response, and deficiency of IL-10 improves wound healing [21,22]. IL-6 levels decreased in response to both treatments (Fig 7C).

### Sircol collagen assay for soluble collagen

**Fig 8:**
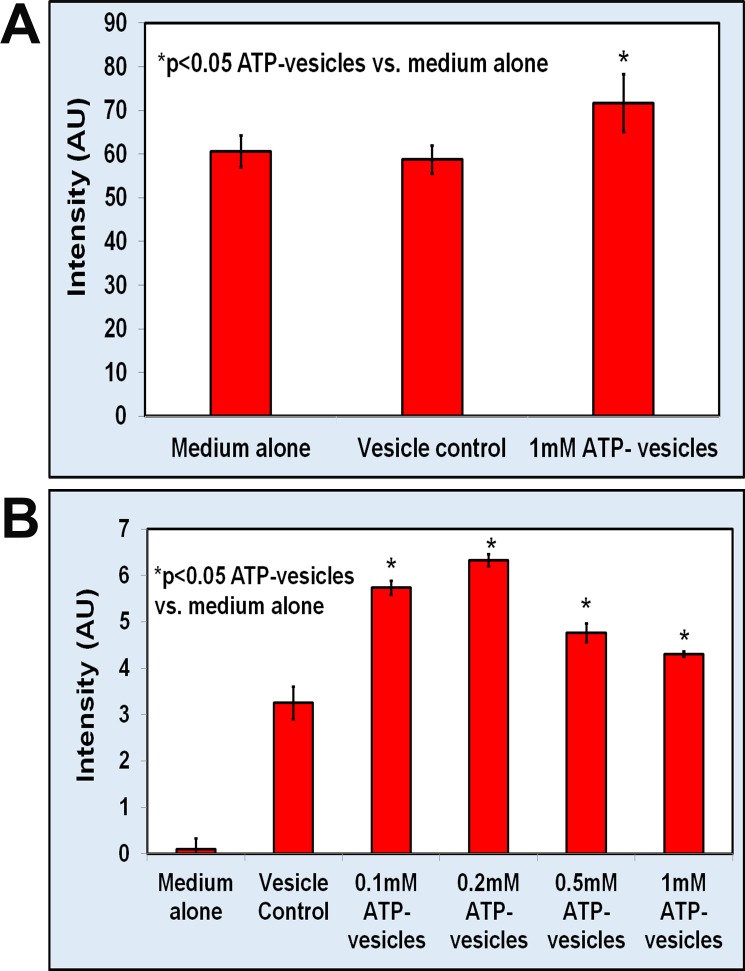
Sircol collagen assay on monocyte/macrophage cells treated with different doses of ATP-vesicles. Soluble collagen production measured by Sircol assay (mean ± SD of six independent experiments, *p<0.05). **(A)**: Human cells. **(B)**: RAW264.7 mouse macrophage cells.

The amount of newly formed soluble collagen produced by the cells was determined using the Sircol collagen assay. We performed this assay in both primary human monocyte/macrophages and in RAW 264.7 mouse cells. In the human monocyte/macrophage culture study, cells (500,000 cells/ml) were cultured under standard conditions and then treated with ATP-vesicles, Regranex, or culture medium 24 h later. Soluble collagen was assayed using Sircol method. It is possible that the process used to isolate the human primary monocyte/macrophages in this study might have stimulated a high basal level of collagen production in these cells. We were able to detect a significant increase in the amount of soluble collagen in the culture supernatant after 48 h of treatment with ATP- vesicles (Fig 8A). The RAW264.7 mouse macrophage cells (500,000 cells/ml) were also cultured under standard conditions and treated 24 h later with different concentrations of ATP-vesicles. The Sircol assays again revealed much higher collagen production in cells treated with various concentrations of ATP-vesicles but the increase was more significant when the Mg-ATP concentration was in the range of 0.1 to 0.2 mM (Fig 8B).

**Fig 9:**
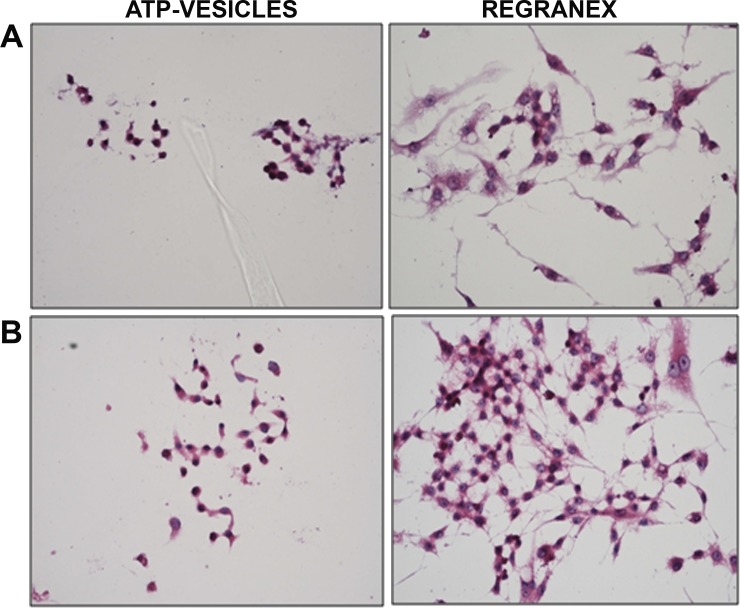
Hematoxylin-eosin staining shows the macrophage morphology in the cells treated with ATP-vesicles. Both the macrophage cells maintain macrophage morphology when treated with ATP-vesicles. **(A)**: Human cells. **(B)**: RAW264.7 mouse macrophage cells.

We also examined whether the cultured macrophages had undergone transformation into fibroblast phenotypes by staining the cells with hematoxylin and eosin and examining them for morphological changes in response to different treatments. Cells treated with ATP-vesicles maintained their original large circular morphology even after 48 h of treatment. The cells treated with Regranex changed their morphology from rounded shapes to elongated fibroblast-like structures (Fig 9A and 9B).

## Discussion

The major findings in this study include the following: 1) intracellular ATP delivery caused rapid tissue regeneration accompanied by early and massive macrophage accumulation that arose as a combination of cell trafficking and in situ proliferation; 2) treatment with ATP-vesicles increased macrophage collagen production whereas the components of ATP-vesicles supplied singly did not show similar effects; 3) macrophage collagen production started very early after ATP-vesicle treatment and continued throughout the study, whereas Regranex treatment increased collagen production only after 48 h of treatment; 4) ATP-vesicle treated macrophages maintained their phenotype and collagen production after 5 d of culture, whereas Regranex treated cells changed their phenotype to fibroblasts after only 48 h of culture; and 5) treatment with ATP-vesicles increased macrophage MCP-1 production but decreased IL-10 production, whereas Regranex treatment produced the opposite effects. The combination of these changes in cell characteristics in response to ATP-vesicles would be expected to result in a very much enhanced healing process in acute wounds.

Among the unprecedented findings was that in situ macrophage proliferation, triggered in the absence of any parasite or bacterial infection, clearly plays a central role in the enhanced healing process. Traditional knowledge holds that progenitor cells and promonocytes proliferate and differentiate in the bone marrow and then enter the circulation, where they mature into monocytes. The monocytes then migrate from the peripheral blood into the tissues, where they transform into macrophages, which are terminally differentiated cells [23,24]. Some localized in situ macrophage proliferation was reported recently during atherosclerosis and adipose tissue inflammation [25–28], this phenomenon has never been reported in healing wounds.

Monocytes are a highly plastic subset of cells in the innate immune system, and they differentiate into macrophages as a result of numerous morphological, biochemical, and functional changes [29]. This differentiation was reported recently in response to infection with the parasite *Litmosoides sigmodontis*, because inflammation or infection results in the release of ATP, which triggers cell mitosis and proliferation [25]. No parasite or bacterial infection was involved in our study, but our PCNA and BrdU immunohistochemistry studies provided clear evidence of active proliferation activity and with quite a dramatic speed and magnitude. Cell proliferation and remodeling both require energy from ATP hydrolysis; therefore, we speculate that some enhancement occurs for the ATPase activities of the BRG1 and Brm subunits of the SWI/SNF complex, which are important for modulation of the chromatin structure that accompanies transcriptional regulation [30–32]. Previous studies have also shown that recruitment of BRG1 mediates T helper cell 2 (TH2) differentiation via IL-4, and this is driven by myeloid colony-stimulating factor (M-CSF) [25,33].

The ATP-dependent remodeling SWI/SNF complex uses the energy of ATP hydrolysis to rearrange chromatin structure—and in doing so, allows transcription of target genes to proceed [34]. Both BRG1 and Brm are ubiquitously expressed in almost all tissues and they participate in cell proliferation. Indeed, our preliminary results showed that BRG1 mRNA levels were 2.1 times higher and Brm mRNA expression was 2.6 times higher in ATP-vesicle treated wounds than in controls. Western blotting indicated that BRG1 protein level was 6.8 times higher and Brm protein expression was 4.8 times higher in ATP-vesicle-treated wounds than in controls. The same results were confirmed by immunohistochemical staining [35].

We also tested the transcriptional regulators, the peroxisome proliferator-activated receptors (PPARs) such as PPARα, PPARβ, and PPARγ as well as PPARγ co-activator 1α (PGC1α), and found increased mRNA expression of these transcriptional coactivators [36]. Still other factors might be involved, but our preliminary results have provided a reasonable explanation for the rapid and massive in situ macrophage proliferation.

The ability of macrophages to produce collagen was proposed two decades ago [37], and collagen production by macrophages is well known to occur in atherosclerosis [38,39], tumor growth and encapsulation [40,41], solid organ fibrosis [42–44], and muscular dystrophy [45]. The possibility of direct collagen production by macrophages in wound healing has also been reported sporadically [46,47]. However, this possibility is still largely dismissed by most wound care specialists because of the lack of direct evidence [37,47,48]. Rapid filling of a large tissue defect requires equally rapid ECM production. Furthermore, the strength of healed wounds is determined by the amount and quality of newly synthesized and cross-linked collagen, as well as by the degradation of provisional extracellular matrix [49,50]. Traditionally, macrophages are not believed to produce collagens, the major structural proteins of the ECM. However, Schnoor et al. [48] demonstrated that macrophages could synthesize and secrete type VIII collagen in vitro. They also reported that macrophages express almost all known collagen and collagen-related mRNAs. Weitkamp et al. [51] reported the in vitro synthesis of type VIII collagen by human macrophages. These subtypes of collagen may play major roles in atherosclerosis, but among the dozens of different types of collagens, the majority (80–90%) of collagen in the skin ECM is composed of subtypes I, II, and III, especially subtype I which accounts for about 80–85% of the total collagen [52].

Collagen type Iα1 is a protein encoded by the COL1A1 gene in humans. It forms the fibrillar collagen found in most connective tissues in the human body. At present, no one has reported direct production of these collagen subtypes by macrophages and especially during wound healing. Numerous cytokines, chemokines, and growth factors have known involvements in wound healing. The classic macrophage activation induced by IFN-gamma is well known, but the alternative pathway induced by IL-4 and IL-13 is relatively less studied. Collagen production by alternative activation is believed to occur through the arginine pathway, which results in proline production—a major component of collagen [53,54]. In fact, arginine administration has been reported to enhance wound healing in various animal models [55,56]. Our results support this possibility and provide a reasonable explanation for the extremely rapid tissue regeneration that occurs when intracellular ATP delivery is used for wound care.

Our previously published in vivo studies clearly indicated the occurrence of a massive macrophage accumulation in the wound site of ATP-vesicle-treated wounds within 24 h of treatment. Thicker fibers and intense staining near the wound site were evident 72 h later by van Gieson staining, indicating enhanced collagen synthesis over that seen in the control wounds treated with normal saline or Regranex [4–6]. We further confirmed the collagen deposition in the wound site by Picrosirius red staining, which, with circular polarized microscopy, showed positive birefringence [57]. The change in birefringence of collagen was three times higher in ATP-vesicle treated wounds than in control wounds. However, collagen production is a complex process that involves cells (fibroblasts, macrophages), as well as other factors such as amino acids, peptides, enzymes, cytokines, and chemokines, which are difficult to differentiate in vivo. Previous studies have shown that exposure of monocytes to the proinflammatory cytokine IL-6 induces monocyte differentiation from a dendritic cell to a macrophage phenotype. Similarly, IL-6 exposure increases the mRNA expression profile associated with M2 macrophages [58,59]. In our studies, the ATP-vesicle-treated monocyte/macrophage cells showed higher IL-6 expression in the first 24 h, suggesting a possible enabling of the macrophage M1 to M2 polarization and subsequent capacity for collagen synthesis. By contrast, higher expression of IL-6 was shown to delay wound healing [60], and this may explain the decrease in IL-6 expression observed after 24 hours.

Numerous cytokines, chemokines, and growth factors have a known involvement in wound healing. Many cytokines have multiple functions and their effects on wound healing are still controversial; therefore, we tested a few of them to expand our efforts to explore the relationship between cytokines and macrophage collagen production. Among the tested cytokines, monocyte chemotactic protein-1 (MCP-1/CCL2) showed significant increases. MCP-1 is the prototype of the CC-chemokine subfamily, and is an IFN-γ-inducible chemokine with multiple functions [61,62]. It is endowed with chemotactic and activating properties for macrophages and other leukocytes, and has a critical involvement in the regulation of inflammatory processes and wound healing. Wood et al. [63], using a mouse model, provided clear evidence that unlike chronic diabetic ulcers, which show a typical persistent and predominant hyper-inflammatory state, early diabetic wounds exhibited a paradoxical and damaging decrease in essential macrophage responses. The resulting reduction in MCP-1 chemokine expression then led to a delayed macrophage response in the diabetic wounds. An increase in MCP-1 also supports the recruitment of additional monocytes by the resident macrophages, whereas a downregulation of MCP-1 inhibits the in vivo recruitment of additional macrophages to wounds [64,65]. At the same time, MCP-1 also acts as a stimulant for M2 macrophage polarization [65,66]. Khan et al. [67] reported that monocyte recruitment in a graded-ischemia wound healing model was severely impaired in MCP-1 knockout mice.

MCP-1 is produced by many cell types, including platelets, endothelial cells, fibroblasts, epithelial cells, smooth muscle cells, monocytes, and microglial cells, but monocytes/macrophages are the major source of MCP-1 [68]. MCP-1 regulates the in vivo migration and infiltration of monocytes, memory T lymphocytes, and natural killer (NK) cells. No one has previously reported the direct involvement of MCP-1 in macrophage collagen production, but its action on fibroblast collagen production has been well studied [69–71]. However, MCP-1 does not have direct effects on dermal fibroblasts due to the lack of functional MCP-1 receptors in these cells. Rather, it triggers the differentiation of interleukin-4 (IL-4)-producing T cells, which then induce collagen synthesis [70]. By contrast, macrophages are rich in MCP-1 receptors (CCR2) and M2 macrophages are known to produce IL-4 [72]. A reasonable assumption, therefore, is that the macrophages have a high capacity for direct collagen production when MCP-1 levels are also high. In our study, the MCP-1 levels were significantly elevated shortly after ATP-vesicle treatment. This phenomenon could be another reason for the extremely early and rapid macrophage accumulation in ATP-vesicle treated wounds because of the reciprocal recruitment [6] MCP-1 is also a strong stimulator of angiogenesis [73,74]. T cells treated with MCP-1 show enhanced production of IL-4 and IL-13 cytokines [75,76] whereas MCP-1 deficient mice cannot increase their Th2 response [77].

Another cytokine that shows significant changes is the potent anti-inflammatory interleukin-10 (IL-10). The impact of the local inflammatory response on the process of wound healing has been debated for decades with respect to whether it promotes or impedes healing. The major sources of IL-10 involved in the wound healing process are still not clear, but macrophages, mast cells, B cells, Th2 cells, regulatory T cells, and keratinocytes are all believed to produce this cytokine [21]. IL-10 was initially considered a cytokine synthesis inhibitory factor that was produced by Th2 cells. Its production, in turn, inhibited cytokine production by Th1 cells reporting to antigen and B cells [78].

Previous studies showed that excessive IL-10 in wounded skin tissue could hamper the recruitment of macrophages to the wound area, thereby delaying wound healing [21]. In support of this idea, IL-10 deficient animals showed enhanced wound healing [22]. The mechanisms are still not established, but one possible explanation is that IL-10 might impede macrophage infiltration, thereby delaying wound healing. Another possibility is that IL-10 could play different roles in different phases of wound healing [21]. However, in our circumstances, any macrophage impediment would definitely delay granulation tissue formation. In our in vitro studies, IL-10 cytokine levels in monocyte/macrophage cells were also significantly lower shortly after the ATP-vesicle treatment when compared to the Regranex-treated cells. This supports our finding that extremely rapid macrophage accumulation and proliferation occur very early.

When we developed the intracellular ATP delivery technique, we tested its true effectiveness by comparing the effects of its individual components (Mg-ATP alone, lipid vesicles alone, and unencapsulated Mg-ATP plus lipid vesicles) on the survival of human umbilical vein endothelial cells (HUVECs) and cardiomyocytes during extreme hypoxia (<0.5% O_2_) or chemical hypoxia (KCN 2 mM). Protection against either form of hypoxia was only achieved with ATP-vesicle treatment [17–19]. We also tested the potential usefulness of the ATP-vesicles in wound care by studying their skin penetration using mammalian skin (rat and pig) and found 10–20 fold increases in ATP uptake when compared with free Mg-ATP [19].

**Fig 10:**
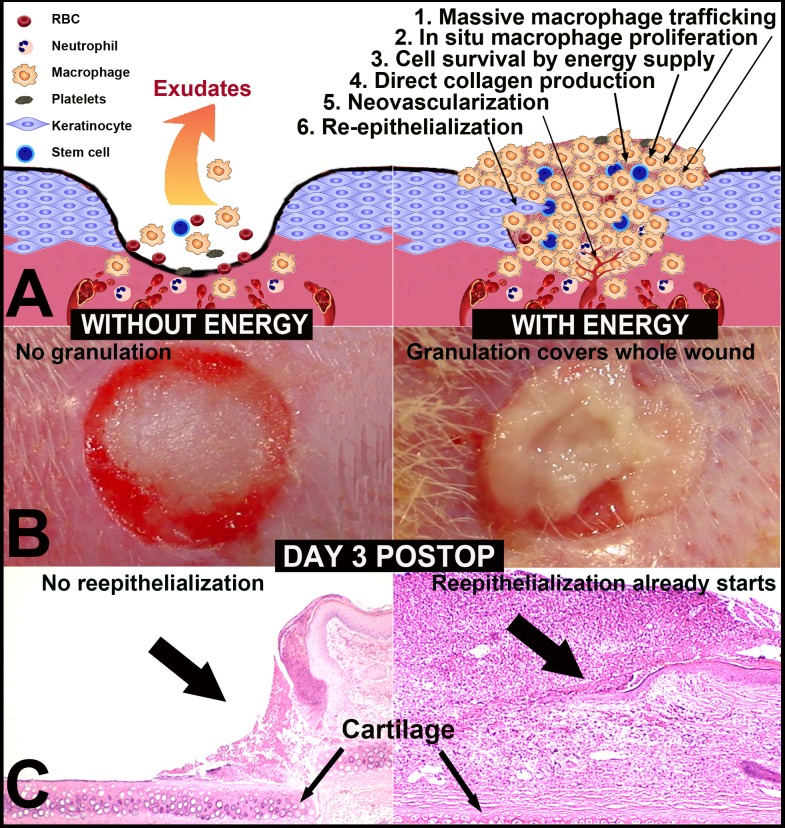
**A schematic illustration of possible mechanisms that could explain the rapid tissue regeneration seen in response to intracellular ATP delivery**: 1) treatment of ATP-vesicles causes massive macrophage accumulation in the wound wall and cavity; 2) the treatment also cause macrophage in situ proliferation; 3) the supplied energy supports cell survival and function in the wound cavity even though neovascularization has not completed; 4) the activated macrophages produce collagen directly; 5) neovascularization is enhanced, which directly transforms the early cell mass into the extracellular matrix; and 6) reepithelialization is enhanced on the basis of rapidly formed granulation tissues. In the control wounds, cell migration and accumulation cannot be achieved because there is no energy supply in the wound cavity.

The unprecedented effectiveness of the ATP-vesicles in promoting wound healing was entirely unexpected—the phenomenon we have reported is unique in wound healing. The very early macrophage accumulation and proliferation cause rapid infilling of the wound cavity, while the direct collagen production by the macrophages results in direct formation of a solid extracellular matrix. The final result is a very early formation of granulation tissue and enhanced healing (Fig 2). The complete mechanisms are probably more complex than the schematic (Fig 10) indicates, but further studies should provide additional useful information that can explain this unprecedented phenomenon in wound healing.

Our current study has the following limitations: 1) One major feature of the ATP-vesicle treatment is the extension of the macrophage phenotype. Treatment with Regranex results in very early transformation from macrophage to fibroblast, within the time frame that fits the normal collagen production process, and this was clearly seen in our in vivo results [6]. At this time, we still do not know the exact mechanisms by which intracellular ATP delivery extends macrophage survival time. 2) Our measured soluble collagen contents are relatively low when compared with some previous reports. This is related to differences in measurement methods and in the numbers of cells used, which complicate side-by-side comparisons with other techniques. The collagen levels in our in vitro studies with primary macrophages also cannot be compared to the in vivo studies due to the lack of T cells in the culture system. Monocyte/macrophage cells need to adopt an alternative pathway for this function and creation of an in vitro microenvironment that mimics that of an in vivo wound to increase collagen synthesis remains an unmet challenge. We envision this to be our first effort to delineate the possible mechanisms, and more studies are needed to fully understand this unprecedented wound healing phenomenon.

In conclusion, intracellular delivery of ATP causes monocytes/macrophages to proliferate in situ and to produce collagen type 1 directly. These responses then lead to rapid tissue regeneration. This result has never been achieved in wounded tissues by any other treatments. We still do not know the exact mechanism by which intracellular ATP delivery extends macrophage survival time or induces their massive in situ proliferation and very early and rapid collagen production, but these responses are highly significant for the treatment of acute and chronic wounds.
